# Mapping the Progressive Treatment-Related Reduction of Active MRI Lesions in Multiple Sclerosis

**DOI:** 10.3389/fneur.2020.585296

**Published:** 2020-11-20

**Authors:** Antonio Giorgio, Marco Battaglini, Giordano Gentile, Maria Laura Stromillo, Claudio Gasperini, Andrea Visconti, Andrea Paolillo, Nicola De Stefano

**Affiliations:** ^1^Department of Medicine, Surgery and Neuroscience, University of Siena, Siena, Italy; ^2^Department of Neurosciences, San Camillo-Forlanini Hospital, Rome, Italy; ^3^Medical Affairs Department, Merck Serono S.p.A., Rome, Italy

**Keywords:** multiple sclerosis, MRI, lesion probability map, brain, white matter

## Abstract

**Objective:** To assess treatment-related spatio-temporal dynamics of active MRI lesions in relapsing-remitting multiple sclerosis (RRMS) patients.

**Methods:** We performed a *post-hoc* analysis of MRI data acquired at weeks 4, 8, 12, and 16, in RRMS patients from the multicenter randomized IMPROVE study, which compares patients treated with 44 mcg subcutaneous interferon β-1a three times weekly (*n* = 120) versus placebo (*n* = 60). We created lesion probability maps (LPMs) of the cumulative combined unique active (CUA) lesions in each patient group at each time point. Group differences were tested in terms of lesion spatial distribution and frequency of occurrence.

**Results:** Spatial distribution of CUA lesions throughout the study was less widespread in the treated than placebo group, with a 50% lower lesion accrual (24 vs. 48 cm^3^/month). Similar results were obtained with the WM tract analysis, with a reduction ranging from −47 to −66% in the treated group (*p* < 0.001). On voxel-wise analysis, CUA lesion frequency was lower in the treated group than the placebo group at week 4 (*p* = 0.07, corrected), becoming particularly pronounced (*p* ≤ 0.03, corrected) from week 8 onwards in large clusters of WM tracts, with peaks along fronto-parietal parts of the corticospinal tract, thalamic radiation, and superior longitudinal fascicle.

**Conclusion:** LPM showed, in the short term, a treatment-related reduction of MRI lesion activity in RRMS patients in specific, clinically relevant brain locations. Such a quantitative approach might be a promising additional endpoint in future MS studies alongside the number and volume of WM lesions.

**Clinical Trial Registration:** ClinicalTrials.gov identifier NCT00441103.

## Introduction

Brain volume and the number of white matter (WM) lesions are commonly used magnetic resonance imaging (MRI) endpoints to test the effect of disease-modifying treatments (DMT) in clinical trials of multiple sclerosis (MS). Generally, however, MRI lesion-based endpoints do not provide information on the specific brain locations where treatment effect may more prominently occur.

Recent clinical studies have used MRI-derived spatial map of WM lesions (i.e., a lesion probability map [LPM]) to assess, at group level, the topographic relevance of WM lesions in terms of lesion distribution and frequency of lesion occurrence in a given brain location. This approach has been applied in cross-sectional studies of several WM disorders including MS (1–13), allowing the capture of spatial patterns of focal pathology not otherwise evident in a single-patient evaluation and also providing clinically relevant information.

We aimed here to apply LPMs in an MS clinical trial to assess topographic differences in lesion distribution and frequency due to treatment effect of a DMT. We used monthly-acquired MRI data (weeks 4, 8, 12, and 16) in relapsing-remitting (RR) MS patients from a multicenter, randomized clinical study (IMPROVE, Investigating MRI Parameters with Rebif imprOVEd formulation) comparing patients treated with 44 mcg subcutaneous (sc) interferon (IFN) β-1a three times weekly (t.i.w.) (*n* = 120) vs. placebo (*n* = 60). LPMs of the cumulative combined unique active (CUA) lesions, a widely used measure of MRI activity in clinical trials, were created for each patient group at each time point. Between-group differences were tested in terms of (i) spatial lesion distribution across the brain and along pre-defined atlas-based WM tracts and (ii) frequency of lesion occurrence across the brain.

## Methods and Materials

### Patient Population

We performed a *post-hoc* analysis of MRI data in RRMS patients from the double-blind phase (16 weeks) of the IMPROVE study, a multicenter randomized (2:1) clinical study (ClinicalTrials.gov identifier NCT00441103) comparing patients treated with sc IFN β-1a 44 mcg t.i.w. (*n* = 120) vs. placebo (*n* = 60).

Study subjects were patients with RRMS (diagnosed according to the McDonald criteria) (14), aged 18–60 years old, with an Expanded Disability Status Scale (EDSS) (15) score ≤ 5.5 at study entry and active disease (i.e., ≥1 clinical event and ≥1 gadolinium [Gd]-enhancing MRI lesion) within the 6 months before randomization. More details on trial design and patient characteristics as well as complete results of the clinical trial are reported elsewhere (16, 17).

### Ethics

This study involving human participants was reviewed and approved by the ethics committees of each center participating in the trial and was conducted in accordance with the Declaration of Helsinki (1996). Prior written informed consent was obtained from all patients.

### MRI Data Acquisition and Analysis

We used MRI examinations (dual-echo, fast spin-echo sequences yielding proton density [PD] and T2-weighted images; T1-weighted images obtained 5 min after injection of Gd; voxel size = 1 x 1 x 3 mm, 44 contiguous [i.e., without gap] slices, axial orientation) acquired at weeks 4, 8, 12, and 16 in order to create, for each patient group, an LPM of the CUA. As in previous studies, a CUA lesion was defined as a gadolinium (Gd)-enhancing T1 lesion and a new or enlarging T2 lesion that does not co-localize with a T1 Gd-enhancing lesion (18).

**Figure 1:**
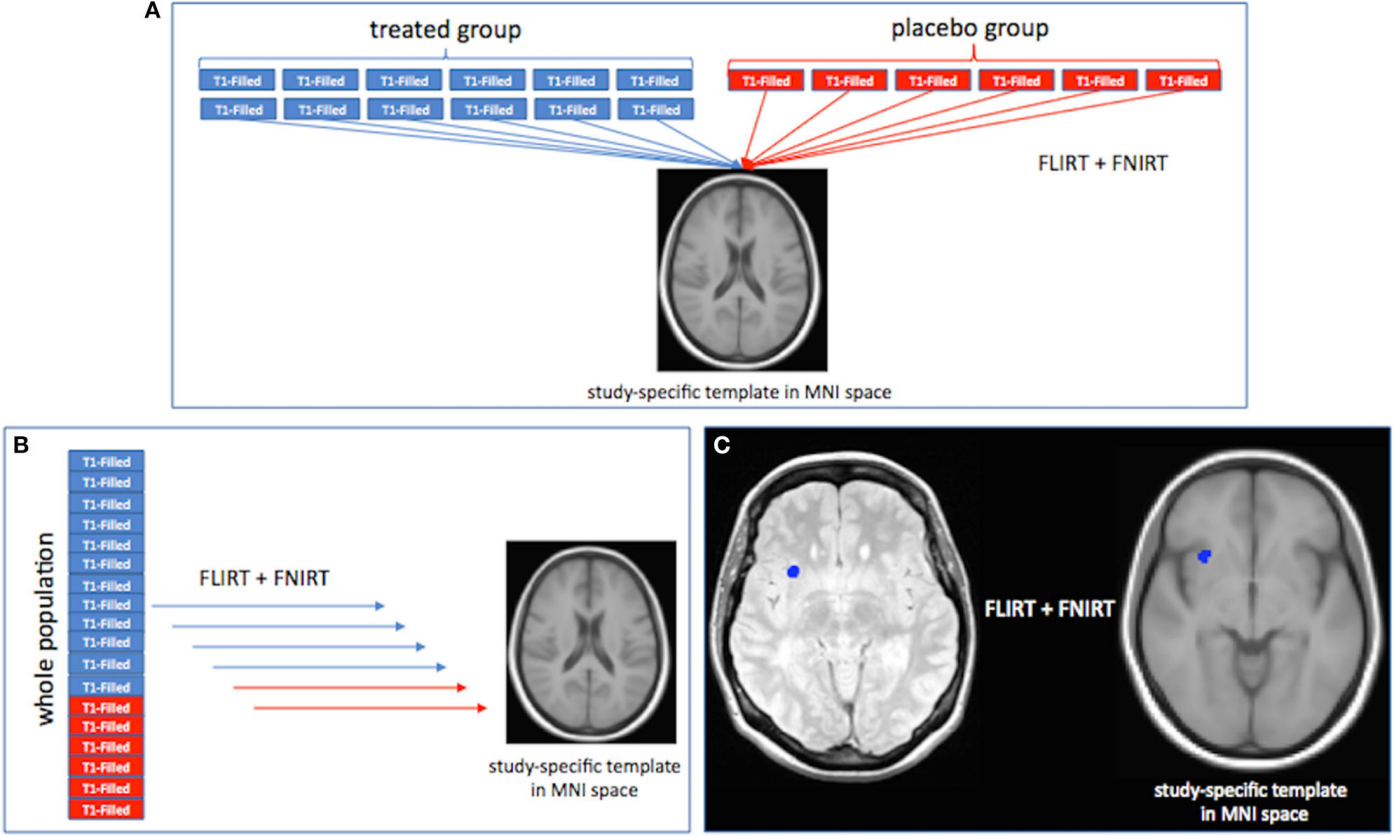
Registration workflow for the creation of the study template in standard space **(A)**, registration onto the template of T1-weighted images, **(B)** and combined unique active (CUA) lesion masks **(C)** of all study patients. See text for details.

LPMs were created using tools from the FMRIB Software Library (FSL) (19, 20). First, a symmetric (i.e., with a balanced number of patients from each group) study template, representative of the whole patient population, was obtained after registering a sample of baseline T1-weighted images (*n* = 60 IFN β-1a and *n* = 60 placebo), previously lesion-filled (21), onto the MNI standard brain (2 mm^3^) by using first linear [the FLIRT (FMIRB Linear Image Registration Tool) (22)], then refinement with non-linear [FNIRT (FMRIB Non-linear Image Registration Tool) (23)] registration, and finally, by merging and averaging all the registered images. Second, T1-weighted images of all study patients were registered onto the study template in standard space using FLIRT and then FNIRT. Third, for all study patients the CUA lesion mask, obtained by the overlap between Gd-enhancing T1-lesion and new or enlarging T2-lesion masks were registered onto the template through FLIRT followed by FNIRT (with nearest neighbor interpolation) using the matrices obtained from previous registrations (i.e., PD onto T1-weighted images with “boundary-based registration” (BBR) cost function, T1-weighted images onto the template). Registration workflow is summarized in Figure 1. Two raters (MB and GG) independently checked all CUA lesion masks registered onto the template, and an agreement was found in all cases. Finally, for each study group (IFN β-1a and placebo) and for each time point (baseline, weeks 4, 8, 12, 16), an LPM was generated by first merging and then averaging all the CUA lesion masks previously registered onto the template. The voxel intensity of the LPM represents the frequency of lesion occurrence in that voxel.

At each time point, differences in lesion spatial distribution between IFN β-1a and placebo groups were assessed first across the brain and then along (un-thresholded for better brain coverage) major WM tracts derived from the Johns Hopkins University (JHU) WM tractography atlas implemented in FSL: thalamic radiations (TR), corticospinal tract (CST), cingulum (Cg), forceps major (Fmaj), forceps minor (Fmin), the inferior fronto-occipital fascicle (IFOF), the inferior longitudinal fascicle (ILF), and the superior longitudinal fascicle (SLF).

Within the general linear model (GLM) framework, we used *randomiz*e, a non-parametric permutation FSL program (*n* = 5,000 permutations) to test comparisons between IFN β-1a and placebo groups in lesion frequency at each time point (baseline, weeks 4, 8, 12, 16) with unpaired *t*-tests and in on-study CUA lesion frequency averaged across follow-up time points (weeks 4, 8,12, and 16) with analysis of variance (ANOVA). The statistical significance level was set at *p* < 0.05, using TFCE (threshold-free cluster enhancement) and correction for multiple comparisons across space. Local maxima were mapped on the JHU WM atlases of tractography and of labels, both implemented in FSL.

## Results

### Baseline Assessment

As mentioned in the previous study (16), patient demographics, baseline disease characteristics, and baseline MRI measures were similar in both patient groups (placebo and treated). In the current study, we also performed a comparison of the baseline LPMs of the two study groups, and no voxel-wise differences in lesion frequency were found (*p* > 0.10, corrected).

### Longitudinal WM Lesion Distribution Analysis

**Figure 2:**
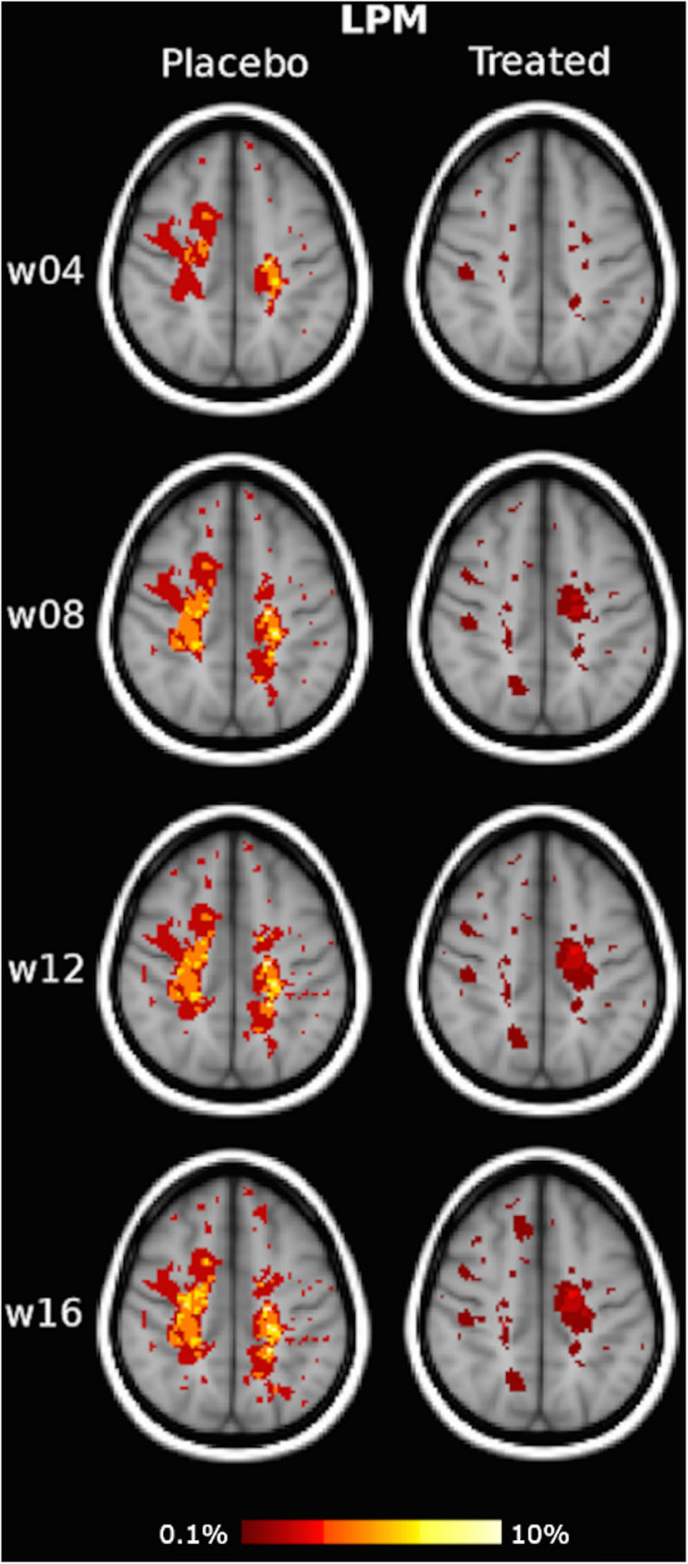
Illustrative slices of lesion probability map (LPM) of the spatial distribution of active MRI lesions, expressed as combined unique active (CUA) lesions, in the placebo and interferon (IFN) β-1a groups at weeks 4, 8, 12, and 16. Red-to-yellow represents the frequency of CUA lesion occurrence (range: 0.1–10%). The background image is the study template in standard space (MNI 2 mm^3^). An animated version of this figure regarding data visualization of the entire brains of each group at each time point is available as Supplementary Material.

Qualitative assessment of LPMs showed that the overall spatial distribution of CUA lesions across the brain was less widespread in the IFN β-1a group than the placebo group at all time points. Indeed, the treated group presented throughout the study an increasing reduction of CUA lesion distribution compared to the placebo group, and this was observed as early as 4 weeks after treatment initiation and reached the maximum at week 16 (Figure 2, Supplementary Video 1).

In terms of quantitative assessment of LPM, the CUA lesion extent of the placebo group was 62 cm^3^ at week 4, 133 cm^3^ at week 8, 168 cm^3^ at week 12, and 196 cm^3^ at week 16, that is, with a mean lesion accrual of 48 cm^3^/month. By contrast, cumulative CUA lesion extent of the treated group was 41 cm^3^ at week 4 (−33% vs. placebo), 76 cm^3^ at week 8 (−43% vs. placebo), 89 cm^3^ at week 12 (−47% vs. placebo), and 95 cm^3^ at week 16, that is, with a mean lesion accrual of 24 cm^3^/month (−50% vs. placebo).

**Figure 3:**
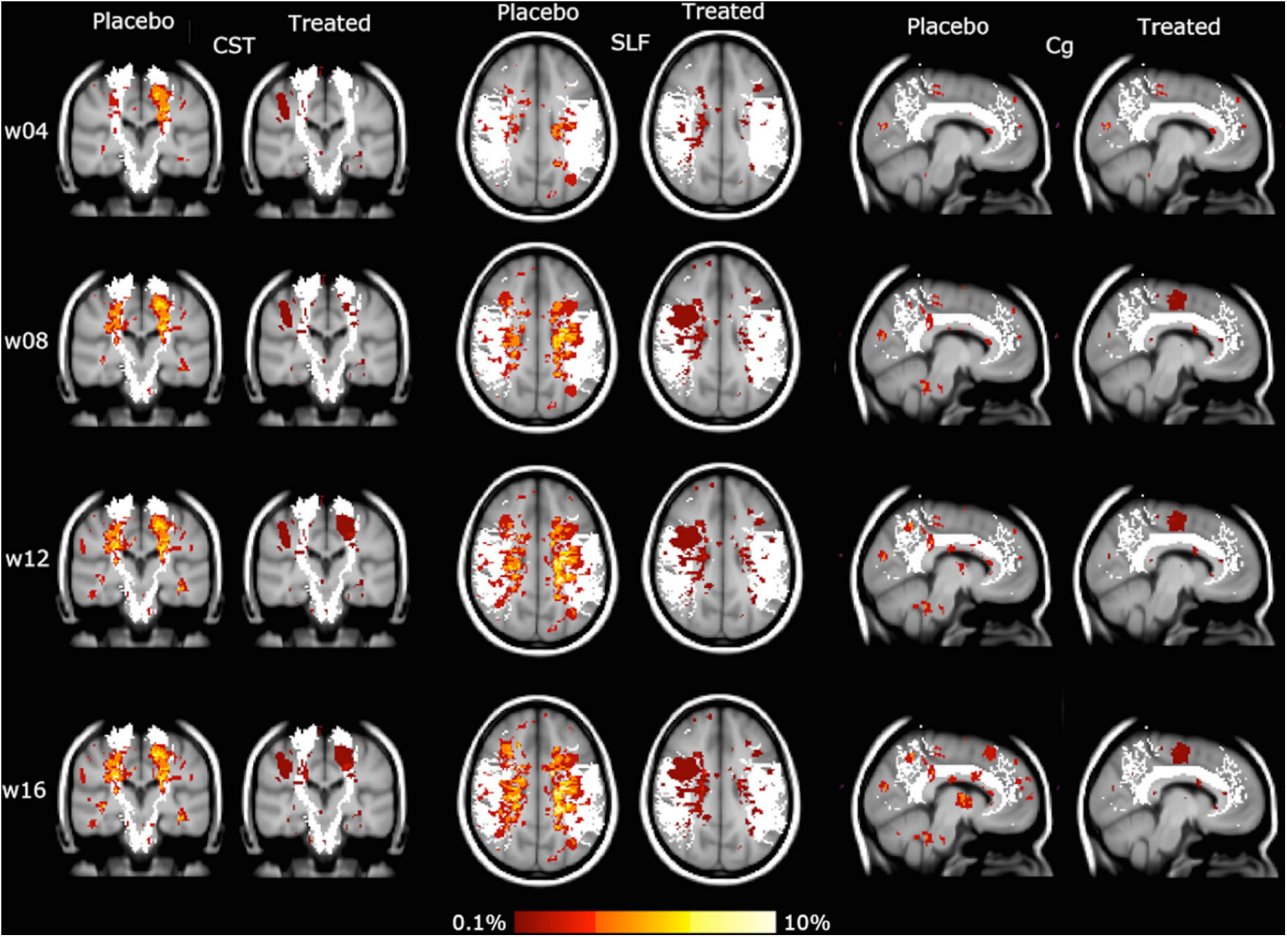
Lesion probability map (LPM) of the spatial distribution of active MRI lesions, expressed as combined unique active (CUA) lesions, mapped on some representative white matter (WM) tracts (in white: corticospinal tract [CST], superior longitudinal fascicle [SLF], cingulum [Cg]). Red-to-yellow represents the frequency of CUA lesion occurrence (range: 0.1–10%). The background image is the study template in standard space (MNI 2 mm^3^).

Similar results were obtained with the WM tract analysis of LPM, with a reduction of CUA lesion accrual in the treated group compared with the placebo group, ranging from −47 to −66% in all the assessed WM tracts of the projection (CST, ATR), association (SLF, IFOF, ILF), and commissural (FM, Fmin, Cg) systems (*p* < 0.001) (Table 1, Figure 3).

**Table 1:** LPM-derived CUA lesion extent along major WM tracts in placebo and treated groups at weeks 8 and 16.

**WM tract(volume, cm^**3**^)**	**Placebo w08 (volume, cm^**3**^)**	**Treated w08(volume, cm^**3**^)**	**Placebo w16 (volume, cm^**3**^)**	**Treated w16(volume, cm^**3**^)**	**Percent difference at w16**	*p*-value at w16^*^
*Projection*						*p* < 0.001
CST (74)	15	5.3	16.7	8.3	−50%	
ATR (102)	11	8.2	20	9.4	−52%	
*Association*						*p* < 0.001
SLF (206)	28.3	20	41.8	22.2	−47%	
IFOF (94)	10	5.8	16	7	−55%	
ILF (96)	8.8	3.8	13.3	4.7	−64%	
*Commisural*						*p* < 0.001
FM (58)	4.8	2.8	8.7	3.7	−56%	
Fmin (51)	3.4	1.7	6.8	2.3	−66%	
Cg (64)	5.7	2	8.4	3.2	−60%	

* *Bonferroni corrected. LPM, lesion probability map; CUA lesion, combined unique active lesion; WM, white matter; CST, corticospinal tract; ATR, anterior thalamic radiation; SLF, superior longitudinal fascicle; IFOF, inferior fronto-occipital fascicle; ILF, inferior longitudinal fascicle; FM, forceps major; Fmin, forceps minor; Cg, cingulum*.

### Longitudinal WM Lesion Frequency Analysis

The highest peak of CUA lesion frequency was mapped in both study groups on the CST at the level of the corona radiata and was on the order of 50% lower in the treated group than the placebo group at all time points: 3.7% vs. 7.5% at week 4, 4.5% vs. 9% at week 8 and 12, 5.3% vs. 9% at week 16.

**Figure 4:**
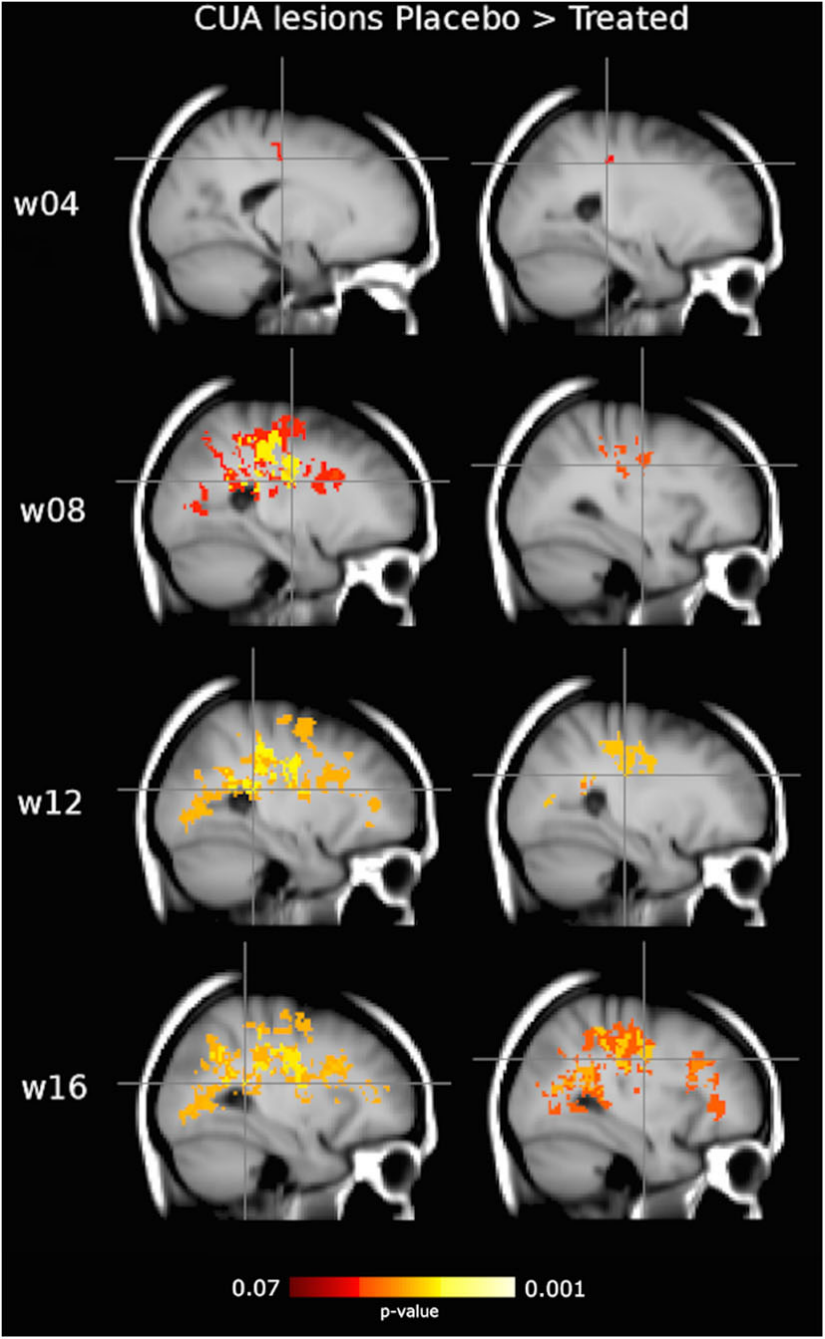
Clusters of active MRI lesions, expressed as combined unique active (CUA) lesions, showing lower frequency in the interferon (IFN) β-1a than placebo group at weeks 4, 8, 12, and 16. Red-to-yellow represents the significance level (*p*-value, range: 0.07–0.001, corrected for multiple comparisons across space). Crosshair points at local maxima, representing the highest reduction in CUA lesion frequency (see Table 2). Background image is the study template in standard space (MNI 2 mm^3^). The most informative slices are shown.

**Figure 5:**
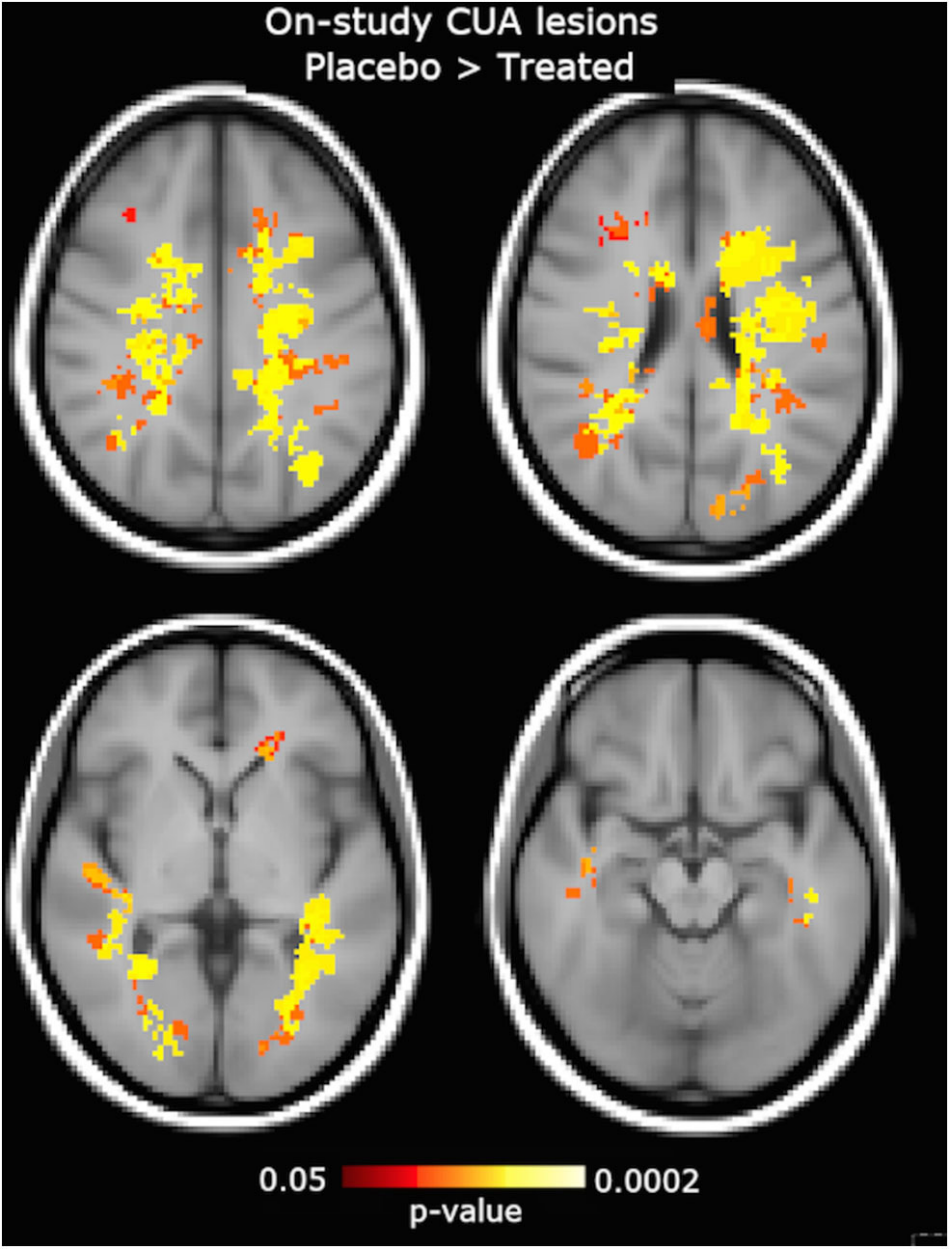
Clusters of on-study active MRI lesions, expressed as combined unique active (CUA) lesions averaged across all follow-up time points (week 4, 8, 12, and 16), showing lower frequency in the interferon (IFN) β-1a than placebo group. Red-to-yellow color represents the significance level (*p*-value, range: 0.05–0.0002, corrected for multiple comparisons across space). The background image is the study template in standard space (MNI 2 mm^3^). The most informative slices are shown.

On voxel-wise analysis across the whole brain, the LPM of the treated group showed lower frequency of CUA lesions than the placebo group since week 4 (*p* = 0.07, corrected) and throughout the study (*p* ≤ 0.03, corrected) in several WM tracts and regions (Figure 4), with local maxima along the fronto-temporal parts of the CST at the level of the corona radiata, TR, and SLF (Table 2). Considering on-study CUA lesions averaged across all follow-up time points (i.e., weeks 4, 8, 12, and 16), widespread WM tracts and regions showed lower lesion frequency in the treated group than in the placebo group (Figure 5).

**Table 2:** Clusters of CUA lesions obtained from voxel-wise analysis across brain showing lower frequency in the IFN β-1a than placebo group at weeks 4, 8, 12, and 16.

**Time point**	**WM tracts/regions (local maxima)**	**Type of WM tract**	**Lobe**	**Side**	**MNI X,Y, Z (mm)**	**Cluster size(voxel count)**	***p*-value (corrected)**
W04	CST/corona radiata	Projection	Parietal	L	−18, −18, 42	35	0.07
	CST/corona radiata (WM of sensorimotor cortex)	Projection	Parietal	L	−24, −32, 40	11	0.07
W08	CST/corona radiata (periventricular)	Projection	Basal ganglia (caudate)	L	−24, −12, 22	6,238	0.002
	SLF (below pre-central gyrus)	Association	Fronto-Parietal	R	32, −10, 32	672	0.03
W12	TR (periventricular)	Projection	Parietal	L	−26, −38, 16	8,174	0.001
	CST/corona radiata (periventricular)	Projection	Parietal	R	28, −24, 26	1,153	0.01
W16	TR (periventricular)	Projection	Parietal	L	−28, −44, 16	8,939	0.003
	SLF	Association	Fronto-Parietal	R	32, −10, 32	7,855	0.01

*CUA lesion, combined unique active lesion; WM, white matter; CST, corticospinal tract; TR, thalamic radiation; SLF, superior longitudinal fascicle*.

## Discussion

By applying a well-established group lesion mapping approach (LPM) to a longitudinal dataset from the double-blind phase (0–16 weeks) of the IMPROVE study, we sought to define for the first time the spatio-temporal dynamics of the evolution of active MRI lesions (measured as CUA) in patients with RRMS treated with IFN β-1a or placebo. The results of the trial have been previously published and demonstrated a rapid and early reduction in the accrual of active MRI lesions in patients with RRMS treated with IFN β-1a (16). In the current study, we found that such short-term effects were clearly reflected at all time points by (i) lower lesion spatial distribution across clinically relevant tracts of the WM associative, projection, and commissural systems and (ii) lower lesion frequency, especially mapping along fronto-temporal parts of WM projection and association tracts, mainly involved in various aspects of motor function.

It is well-known that the pathophysiology of MS lesions is dynamic and changes over time. Nowadays, MRI turns out to be the best tool for tracking the evolution of MS lesions and evaluating treatment response. The strength of a quantitative MRI approach such as LPM, applied here for the first time to a longitudinal dataset, is the ability to reveal, at group level, spatio-temporal patterns of focal WM pathology that, in this context, would not be evident from serial MRI scans of single patients.

Findings of the current study clearly showed a reduction already occurring at week 4 in the spatial distribution of active MRI lesions, as evaluated both qualitatively and quantitatively. As for the latter, the extent of active MRI lesions across the brains of the treated group was, compared with the placebo group, already one-third at week 4 and progressively decreased throughout the study before reaching a level of 50% less at the end of the evaluation (week 16). These results were confirmed by also considering the LPM-derived spatial distribution of active MRI lesions along atlas-based WM tracts. Indeed, the reduction of lesion accrual was extensively and significantly present in all evaluated major WM tracts of projection (CST, ATR), association (SLF, IFOF, ILF) and commissural (FM, Fmin, Cg) systems, ranging from −47 to −66% (Table 1).

In terms of the frequency of occurrence of active MRI lesions, the peak (i.e., the highest probability of finding a lesion) at all time points of both study groups mapped along the CST at the level of the corona radiata, where the treated group showed reductions on the order of 50% compared with the placebo group starting week 4 and throughout the study.

In addition, by performing a voxel-wise analysis across the whole brain, the treated group showed a lower frequency of active MRI lesions than the placebo group at all time points. In particular, this difference was already present, with marginal significance, at week 4 but then became more evident in the subsequent time points (i.e., weeks 8, 12, and 16), involving very large WM clusters. In particular, the highest effect within such clusters mapped along the CST at the level of the periventricular corona radiata of the parietal lobe, along the periventricular TR in the parietal lobe, and along the SLF of the fronto-parietal lobe. This is particularly interesting since (i) CST is the most relevant descending projection tract linking motor cortex to spinal cord and the most clinically eloquent tract for ambulation, which represents the major contributor to physical disability in MS; (ii) TR, also known as the thalamo-cortical pathway, is also a projection tract and links, through the internal capsule, thalamic nuclei with anterior sensorimotor cortex and with posterior visual and auditory cortices; and (iii) SLF is a type of association tract, mediating intra-hemispheric long-range connectivity, linking various regions of parietal and frontal cortices and playing a role in the regulation of the higher aspects of motor behavior.

The strength of the current study lies in the use of the MRI-derived LPM, which, by avoiding the biases of pre-defined regions of interest and WM lesion grouping and taking into account the complexity of WM anatomy, evaluates *in vivo* the anatomical distribution and frequency of occurrence across all WM lesions. In recent years, LPM has been exclusively applied to cross-sectional datasets in various settings: predicting conversion of clinically isolated syndrome (CIS) to MS (9); assessing spatial relationships with diffusion tensor imaging-derived microstructural damage along WM tracts (24, 25), with “patterns” of GM and WM damage (13); assessing cognitive relevance in MS (8) and mild vascular cognitive impairment (11); and distinguishing between: CIS and migraine with aura (12), MS and *neuromyelitis optica* spectrum disorder (10), MS and radiologically isolated syndrome (7), pediatric- and adult-onset MS (26), RR and primary progressive MS in terms of cortical lesions (6).

Some methodological aspects of the study are worthy of mention. First, the very short time frame of the study (i.e., 16 weeks) should have prevented significant differences in atrophy during the follow-up period, as previously reported on the same patient population (16, 17). Given this, we would conclude that it is very unlikely that the very large difference found in CUA lesion frequency and extent over time between the placebo group and the treated group could be influenced at all by differences in brain atrophy. Second, the short time of the study (i.e., 16 weeks) did not allow a reliable assessment of the clinical relevance of the proposed method, which is beyond the scope of this study, and longer studies are needed to explore this important aspect.

In conclusion, the availability of a longitudinal multi-time point MRI dataset allowed us to apply LPM for the first time in the assessment of the spatio-temporal dynamics of MS lesions in the short term. Indeed, we found that rapid and early treatment-related reduction of active MRI lesions mainly mapped along “strategic” regions of WM projection and association tracts located in the fronto-parietal lobe and meaningful for various aspects of motor function. On the basis of our findings, we believe that LPM can complement the commonly used MRI endpoints of lesion changes in clinical trials, providing clinically relevant information on treatment effect in specific brain locations.

## Data Availability Statement

The raw data supporting the conclusions of this article will be made available by the authors, without undue reservation.

## Ethics Statement

The studies involving human participants were reviewed and approved by the Ethics Committee of each participating center, and was conducted in accordance with the Declaration of Helsinki (1996). The patients/participants provided their written informed consent to participate in this study. Full list of all Ethics Committee names/institutions are available in Supplementary Material.

## Author Contributions

AG: analysis and interpretation of data for the work, drafted the work, final approval of the version to be published, and agreement to be accountable for all aspects of the work. MB and GG: analysis and interpretation of data for the work, revision of the work critically for important intellectual content, final approval of the version to be published, and agreement to be accountable for all aspects of the work. MS and CG: acquisition and analysis of data for the work, revision of the work critically for important intellectual content, final approval of the version to be published, and agreement to be accountable for all aspects of the work. AV and AP: revision of the work critically for important intellectual content, final approval of the version to be published, and agreement to be accountable for all aspects of the work. ND: substantial contributed to the conception and design of the work, interpretation of data for the work, drafted the work, revision of the work critically for important intellectual content, final approval of the version to be published, and agreement to be accountable for all aspects of the work. All authors contributed to the article and approved the submitted version.

## Conflict of Interest

CG received a fee as speaker or advisory board by Teva, Novartis, Roche, Merck KGaA, Bayer, Almirall, and Biogen. AV and AP were employees of Merck Serono S.p.A., Rome, Italy, an affiliate of Merck KGaA, Darmstadt, Germany. NDS was a consultant for Biogen, Merck KGaA, Novartis, Roche, Sanofi-Genzyme, Schering, and Teva; and had grants or grants pending from FISM and Novartis, was on the speakers bureaus of Biogen, Teva, Novartis, Sanofi-Genzyme, Roche, and Merck KGaA; had received travel funds from Merck KGaA, Novartis, Roche, Sanofi-Genzyme, and Teva. The remaining authors declare that the research was conducted in the absence of any commercial or financial relationships that could be construed as a potential conflict of interest. The Handling Editor declared a past co-authorship with one of the authors CG.

## Supplementary Material

Supplementary Video 1 A movie loop of the lesion probability map (LPM) of combined unique active (CUA) lesions at weeks 4, 8, and 16 in the placebo and interferon (IFN) β-1a groups.
