# Supplementary material for: Mapping the Progressive Treatment-Related Reduction of Active MRI Lesions in Multiple Sclerosis
Source: Front Neurol. 2020 Nov 20;11:585296. doi: 10.3389/fneur.2020.585296 (PMC7714945; doi:10.3389/fneur.2020.585296)
Supplement: Supplementary file 1 [file Data_Sheet_1.DOC]

| **Institution/ Facility name** | **City** | **Country** |
| --- | --- | --- |
| UMBAL "St.Marina" Clinic of Neurology | Varna | Bulgaria |
| SBALNP - "St. Naum" EAD 3-rd Clinic of Neurology | Sofia | Bulgaria |
| UMBAL "Alexandrovska" Clinic of Neurology | Sofia | Bulgaria |
| SBALSSZ NKB - Sofia EAD Neurology Department | Sofia | Bulgaria |
| UMBAL Pleven 1st Clinic of Neurology | Pleven | Bulgaria |
| Medical Institute - Ministry of Interiority Neurology Department | Sofia | Bulgaria |
| UMBAL "St. Georgi" Clinic of Neurology | Plovdiv | Bulgaria |
| CHUM Hôpital Notre-Dame | Montréal | Canada |
| The Ottawa Hospital General Campus | Ottawa | Canada |
| University of Alberta | Edmonton Alberta | Canada |
| West-Tallinn Central Hospital Centre of Sclerosis Multiplex | Tallinn | Estonia |
| Tartu University Clinics Dept of Neurology & Neurosurgery | Tartu | Estonia |
| Universitätsklinikum Ulm Dept. of Neurology at RKU | Ulm | Germany |
| Universita degli Studie di Genova Dipart. Di Sc. Neurologiche e di Neuroriabilitazione | Genova | Italy |
| Ospedale San Camillo | Roma | Italy |
| Vilnius university Hospital Santariskiu Clinics | Vilnius | Lithuania |
| Colentina Clinical Hospital Clinica de Neurologie | Bucharest | Romania |
| Institute of Human Brain, RAS | St Petersburg | Russia |
| Moscow State Institution of Health Care  «City Clinical Hospital # 11» of Moscow Board of Health | Moscow | Russia |
| State institution «Science research institute of clinical and experimental lymphology of Russian Academy of Medical Sciences (Siberian branch)» | Novosibirsk | Russia |
| State educational institution of the higher professional education of the Ministry of Health of Russian Federation «Samara State Medical University» | Samara | Russia |
| Clinical Center of Serbia, Institute of Neurology | Belgrade | Serbia |
| Clinical Center "Kragujevac", Department of Neurology | Kragujevac | Serbia |
| Hospital Xeral Cíes Servicio de Neurologia | Vigo - Pontevedra | Spain |
| Hospital Clinic i Provincial Servicio de Neurologia | Barcelona | Spain |
| Hospital Central de Asturias Servicio de Neurologia | Oviedo - Asturias | Spain |
